# Impact of the COVID-19 Pandemic on Blood Transfusion among Hospitalized Patients with Chronic Kidney Disease

**DOI:** 10.3390/medicina60091512

**Published:** 2024-09-17

**Authors:** Sevigean Ali, Mihaela Botnarciuc, Iulia-Andreea Badea, Andreea Alexandru, Liliana-Ana Tuta, Lavinia Carmen Daba, Leonard Gurgas, Sergiu Ioachim Chirila

**Affiliations:** 1 Preclinical Disciplines Department, Faculty of Medicine, Ovidius University of Constanta, Campus B, Aleea Universitatii nr. 1, 900470 Constanta, Romania; mihaela.botnarciuc@univ-ovidius.ro (M.B.); iulia.badea@365.univ-ovidius.ro (I.-A.B.); lavinia.daba@univ-ovidius.ro (L.C.D.); leonard.gurgas@365.univ-ovidius.com (L.G.); sergiu.chirila@univ-ovidius.ro (S.I.C.); 2 Blood Transfusions Unit, Emergency Clinical County Hospital Constanta, Bdul Tomis nr. 145, 900591 Constanta, Romania; 3 Nephrology Department, Emergency Clinical County Hospital Constanta, Bdul Tomis nr. 145, 900591 Constanta, Romania; alexandra_med16@yahoo.com; 4 Clinical Medical Disciplines Department, Faculty of Medicine, Ovidius University of Constanta, Campus B, Aleea Universitatii nr. 1, 900470 Constanta, Romania

**Keywords:** transfusion, COVID-19, chronic kidney disease, mortality

## Abstract

*Background and Objectives*: Hematological disorders, especially chronic anemia and coagulation disorders, are common in patients with chronic kidney disease (CKD). Severe anemia is associated with increased cardiovascular morbidity and mortality in this special group of patients and is also responsible for decreased hope and quality of life. Despite the use of appropriate iron therapy and erythropoietin-stimulating agents, red blood cell transfusion is occasionally required, usually in the setting of acute bleeding or for correction of perioperative anemia. The COVID-19 pandemic has accelerated the progression of chronic diseases and worsened the outcomes for patients with nephrological conditions. As a precautionary measure against infections, patients’ access to hospitalization for their procedures has been reduced and their chronic complications, including hematological abnormalities, have gotten out of control. *Materials and Methods:* Our retrospective observational study was designed to evaluate the impact of the COVID-19 pandemic on blood transfusion for the patients with chronic kidney disease hospitalized in our emergency county medical unit, over a period of four years (2019–2022) who were admitted or at least referred for evaluation to the Nephrology department. We also followed the measures adopted to ensure the necessary blood products during this time. *Results:* Between 2190–2022, a total of 24,096 hospitalized patients were transfused at the Emergency County Clinical Hospital in Constanta, Romania. Meanwhile, in the nephrology and other medical or surgical wards of our medical unit, 1590 CKD patients were transfused with different blood derivatives. During the pandemic years, as expected, the number of transfused patients and transfused blood units decreased by 4% and 7%, respectively, in comparison with the pre-pandemic year, 2019. Unlike the general trend of transfusion activity, more patients with CKD transfused in 2022 (580) than before the pandemic (414 in 2019), and the number of blood units was higher in 2022 than in 2019 for red blood products and plasma. Between 2020–2022, from the total number of transfused patients in our study, 254 with CKD patients (16%) and 798 non-CKD (4%) died in-hospital. *Conclusions:* The adaptive strategies implemented to ensure the necessary blood products in the hospital during the COVID-19 pandemic mainly included restrictive transfusion and limitation of elective surgical procedures. The subject matter of the article is important as blood shortages are a problem that healthcare workers may encounter in future pandemics.

## 1. Introduction

Renal patients, especially those with moderate-to-severe chronic kidney disease (CKD) are prone to hematological abnormalities, especially severe anemia, mainly due to the reduced secretion of erythropoietin and impaired erythrocyte survival in the uremic toxic environment. Severe anemia is associated with decreased cardiac function and increased cardiovascular morbidity and mortality in CKD patients and is also responsible for decreased hope and quality of life [1]. The best options in these situations are the correction of iron deficiency and administration of human recombinant erythropoietin or newer drugs like hypoxia-inducible factor-prolyl hydroxylase inhibitors (HIF–PHIs) [2].

Since the discovery of erythropoietin stimulating agents (ESA) and the use of appropriate intravenous iron therapy, red blood cells transfusion is only occasionally required. But in CKD patients and end-stage kidney disease (ESKD), especially for those on hemodialysis, with severe anemia and acute, active bleedings, blood transfusions remain the life-saving option. However, blood transfusion is limited by the availability of blood products, depending on blood donations that were considerably reduced during COVID-19 pandemics [3].

Patients with ESKD treated by chronic dialysis are more likely to need blood transfusions, especially those performing hemodialysis (HD) than those on peritoneal dialysis (PD) therapy because of the HD procedure itself. Patients on hemodialysis are losing blood due to hemolysis, trapped blood in the dialyzer, and an increased risk for gastrointestinal bleeding, caused by antiaggregant or anticoagulant used for their cardiovascular complications. However, aggressive iron replacement and ESAs has largely eliminated the need for red blood cell transfusions, even for patients on HD therapy [4].

Another hematological abnormality described in CKD patients, especially in those with nephrotic syndrome is the excessive clotting and thrombocytopenia with associated bleeding due to platelet dysfunction, mediated by uremic toxins [5]. Main pathogenic mechanisms of thrombosis in CKD patients are platelet activation and the occurrence of platelet-leukocyte conjugates and platelet-derived microparticles [6].

Access to safe access remains a luxury for many in the realm of blood products. In the majority of low- and middle-income countries, efforts to ensure safe blood are hindered by low donation levels [7] and limited availability of blood testing equipment [8]. Globally, approximately 42% of blood is collected in high-income countries, which paradoxically house only 16% of the global population [9].

Safe and adequate blood supply can only be guaranteed through regular donations from unpaid, voluntary blood donors [10]. In many low- and middle-income countries, there is a critical shortage of blood available for transfusion [11]. This shortfall has devastating consequences, contributing to morbidity and mortality due to complications of chronic kidney disease, trauma, obstetric hemorrhage, pediatric anemias, and other conditions [11]. The process from blood collection from a donor to its administration to a patient involves numerous aspects, including donor availability, blood processing, and delivery [12]. Thus, it is crucial to optimize existing strategies and introduce new approaches to ensure safe and adequate blood supply worldwide [11].

Although the primary route of transmission of the SARS-CoV-2 virus is through respiratory droplets, research has indicated that viral RNA can be detected in blood samples, raising concerns regarding the safety of blood donations and blood-derived products [13].

There are a lot of studies that have described the impact of COVID-19 pandemic upon blood donations in different regions of the world [14,15]. Therefore, in this article, we aim to analyze the effect of the pandemic blood administration by comparing transfusable blood units. This study was performed for patients hospitalized in emergency medical unit for urgent or critical conditions, who already had chronic kidney disease and needed blood derivatives transfusion to control severe anemia and/or thrombocytopenia, either as complications of the renal disease or other comorbidities, like severe infections or autoimmune diseases.

## 2. Materials and Methods

### 2.1. Study Design and Participants

We conducted a retrospective observational study on a population of patients with variable degrees of chronic kidney disease who needed blood transfusions and derivatives during their hospitalization at our emergency clinical county hospital in the South-Eastern Romania.

Our study group included adult patients (≥18 years old) already diagnosed with CKD in the pre-pandemic year 2019, as well as those admitted between February 2020 (when COVID-19 epidemic started in Romania) and December 2022. After WHO declared COVID-19 a Public Health Emergency of International Concern (PHEIC), and later a pandemic on 11 March 2020, patients were tested for viral infection. Patients admitted in the study were monitored until the completion of their hospitalization (discharged or dead).

The exclusion criteria were age <18 years, lack of consent to participate in the study, presence of severe heart and hepatic diseases, neoplasia (irrespective of localization) or incomplete data in the medical records.

Clinical charts and hospital electronic records were used as sources of data. We utilized blood transfusion service records, and blood demand and issuance from our blood transfusion unit in the Emergency County Clinical Hospital of Constanta. The study was approved by the local ethics committees and was in accordance with the 1976 Declaration of Helsinki and its later amendments. 

### 2.2. Variables (Outcome and Exposure)

CKD was diagnosed according to the 2012 KDIGO guidelines, consisting in renal morphological, functional and imagistic abnormalities, or an eGFR < 60 mL/min/1.73 m^2^ lasting for more than three months, thus identifying five groups according to the stage of disease: eGFR < 15 mL/min/1.73 m^2^ (stage G5), 15–29 mL/min/1.73 m^2^ (stage G4), 30–59 mL/min/1.73 m^2^ (stage G3), 60–89 mL/min/1.73 m^2^ (stage G2), and >90 mL/min/1.73 m^2^ (stage G1) [1]. We included in our study only patients G3–G5 pre-dialysis, already confirmed with CKD, as well as patients under substitution of renal functions, meaning patients on chronic dialysis or with a functional kidney transplant, for at least one year after the procedure.

A diagnosis of diabetes was made according to the American Diabetes Association (ADA) and the most recent guidelines based on the clinical history of the patient and laboratory exams at admission [16]. A diagnosis of hypertension was made according to the most recent guidelines and the patient’s medical history [17].

Pre-transfusion tests were performed by determining blood type using two complementary methods: the Beth Vincent method (globular) and the Simonin method (serum), the determination of Rh and Kell phenotypes by gel column agglutination technique. The irregular antibodies test [IAT] was conducted using the gel column agglutination technique with LISS/Coombs and Enzyme [Bio-Rad] cards, which can detect IgG class antibodies against Rh, Kell, and Kidd antigens.

Direct compatibility tests [crossmatch], including the saline test, enzyme test, and Anti-Human Globulin [AHG] test were conducted using the gel column agglutination technique, between the recipient’s serum and the red blood cells from each blood bag intended for transfusion.

Diagnosis of SARS-COV two infection was made by RT-PCR method (Real-Time Polymerase Chain Reaction) using ExiPrep™16 Dx by BIONEER (Oakland, CA, USA). For this purpose, nucleic acid extraction was accomplished by the work of four automatic devices available at the St. Andrew’s Emergency Clinical County Hospital’s Molecular Biology Laboratory of Constanta. The estimate time for completing the process is approximately 60 min. The amplification step was performed on a CFX 96 termocycler. The kits of reagent detect the presence of the genes RDRP N, and E.

The exposure variables were collected during hospital admission as follows: (a) demographic and anthropometric characteristics (sex, age, previous medication, and real-time PCR swab specimen); (b) previous comorbidities (smoking habit, diabetes, hypertension, chronic cardiac disease, CKD, gastro-intestinal diseases, or malignancies); (c) main laboratory data (creatinine and eGFR calculated by the CKD-EPI equation); and (d) number and types of blood derivatives transfused per patient and per year.

Indications for red blood cell concentrate (PRBC) transfusion were in patients with active or acute bleeding and patients with anemia-related symptoms like tachycardia, dyspnea on exertion, precordial pain, and hemoglobin less than 8 g/dL [18].

Platelet concentrates were used mainly for the treatment of severe thrombocytopenia, which may occur in CKD patients with drug-induced bone marrow failure, and other immune and nonimmune causes of platelet destruction, like sepsis and disseminated intravascular coagulation [19].

Fresh frozen plasma transfusion was used for the CKD patients undergoing urgent surgery or invasive procedures in the presence of abnormal coagulation tests, the reversal of acenocoumarin in the presence of active bleeding, cases of thrombotic microangiopathy, and congenital or acquired factor deficiency with no alternative therapy [20].

### 2.3. Statistical Analysis

Data were assigned in order to divide the enrolled population into groups based on blood component necessity—red blood cell concentrate (PRBC), platelet (PLT) concentrate, or fresh frozen plasma (FFP). The variables included age, sex, chronic kidney disease, diabetes, hypertension, hyperlipidemia, cardiac diseases, urea and creatinine levels, and platelet count.

Categorical data were collected and organized into a database using Microsoft Office Excel 2016. Descriptive and inferential statistical analyses were performed using GraphPad Prism version 8.4.3. Categorical variables were illustrated in graphics and tables as absolute values or percentages and were analyzed using Fisher’s exact test. Continuous data were described by mean and standard deviation. The Mann–Whitney U test was used to compare differences regarding the total number of transfusions between the non-pandemic year 2019 and years 2020, 2021, and 2022. We also compared the number of transfusions in the chronic kidney disease patients using the Mann-Whitney U test. A *p* value of <0.05 was considered statistically significant.

## 3. Results

We enrolled 1590 hospitalized patients with already diagnosed chronic kidney disease (stage G3 to G5, eGFR < 60 mL/min/1.73 m^2^), without previous known risk factors for severe anemia (gastrointestinal diseases, neoplasms, or hematological malignancies), representing 6.5% of the total number of 24,096 hospitalized patients that received blood derivative transfusions in the same interval of time.

Of the CKD 1590 patients included in the study, the mean age was 62.8 years (SD 14.87), and the majority were females (62.2%).

The study population was stratified according to the stages of eGFR (G3, G4 and G5— pre-dialysis, with kidney transplantation (G3-5T), or on dialysis (5-HD or 5-DP)). We must mention that *p*-values were not calculated for age, creatinine, and eGFR because these variables were already considered during the eGFR measurement. All demographic and clinical characteristics at inclusion in the study group are reported in Table 1.

**Figure 1:**
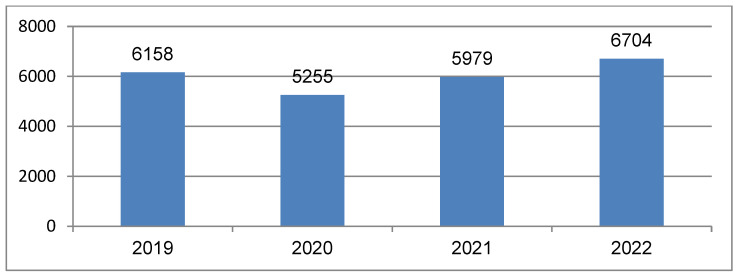
The total number of patients transfused per year between 2019 and 2022.

Regarding the total number of transfused patients, compared to 6158 patients in 2019, a decrease was noted in 2020 (*p* = 0.01) due to the COVID-19 pandemic and the limitation of hospital admissions. Then, in 2021, the number of transfusions slightly increased compared to 2019 (*p* = 0.01), but no significance between 2021 and 2020 was shown (*p* = 0.34). After the COVID-19 restrictions were lifted, in 2022 there was an increase in total transfusions (*p* = 0.03), but we found no statistical significance between 2022 and 2019 (*p* = 0.16), as seen in Figure 1.

Before the pandemic, in 2019, 6158 patients were transfused, receiving a total of 1195 erythrocyte concentrate, representing a percentage of 54%; 6558 units of fresh frozen plasma, representing 30%; and 3445 units of platelets, representing 16%. In 2020, the pandemic year, a decrease in the number of transfused patients was observed, namely 5255. A number of 9391 units of erythrocyte concentrate were administered, representing a percentage of 55%. A similar percentage to that registered in 2019 was recorded for the number of units of fresh frozen plasma (30%) and platelet units (15%) (Table 2).

During the 2020 pandemic year, we observed a decrease to 300 of the number of transfused CKD patients (*p* = 0.01).

In the following pandemic year, 2021, the number of transfused patients was relatively similar to year 2020, with 5979 patients. We noticed a slight increase of 724 transfused patients, but no statistical significance was found (*p* = 0.14). Likewise, in the total number of units transfused, we had a percentage equal to that of 2020: a percentage of 56% units of erythrocyte concentrate, 29% representing 5154 units of fresh frozen plasma transfused, and the number of platelets remained the same as in 2020. For patients with chronic kidney disease, it was not a significant difference between 2021 and 2020 transfusions (*p* = 0.09).

In 2022, there was a significant increase in the number of transfused patients compared to previous years (*p* = 0.001). It can be assumed that 2022 was also the year with the most transfused patients, representing the highest percentage in these four years at 57%. We can also see the number of transfused units included 11,020 units of erythrocyte concentrate, 5843 units of fresh frozen plasma, and 2593 platelet units. We can say that also in the case of patients with chronic kidney disease, there was a significant increase of 580 patients with chronic kidney disease, making 2022 the year with the most transfused units, with a mean of 3 units/patient (range 1–10).

Regarding the type of blood products administered, in the pre-pandemic year, 2019, 283 patients with chronic kidney disease received erythrocyte concentrate, 107 patients received fresh frozen plasma, and 24 patients were transfused with platelet concentrates (Table 3).

**Figure 2:**
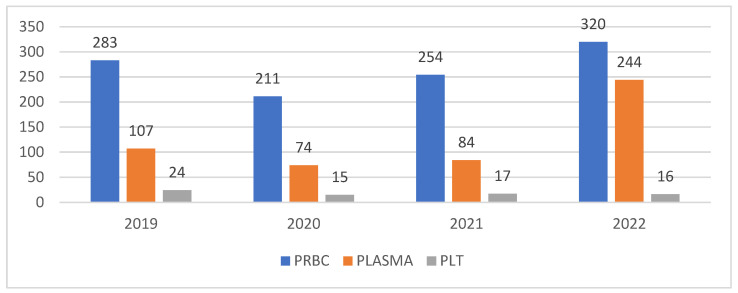
The total number of patients transfused with chronic kidney disease.

In 2020, we observed fewer CKD patients transfused with erythrocyte concentrate, with 211 patients transfused compared to 283 in 2019. We registered a decrease in patients who received fresh frozen plasma (74 patients), and the number of patients who received platelets was only 15. In 2021, we noticed an increase in the number of transfused patients: 254 patients received erythrocyte concentrate, 84 patients received FFP, and 17 patients received PLT concentrates (Figure 2).

In 2022, we noticed an increase to pre-pandemic levels of the transfused patients, with 320 patients receiving erythrocyte concentrate, 244 patients receiving freshly frozen plasma, and 16 patients receiving platelet concentrates.

We monitored for our study group differences regarding PRBC transfusion units between patients with and without chronic kidney disease, and the results are presented in Table 4.

Regarding PRBC transfusions, a positive association with CKD was noted in 2019 (*p* = 0.004, OR 1.23), 2020 (*p* = 0.001, OR = 1.4), and 2021 (*p* = 0.001, OR 1.41). However, in 2022, a negative association was observed (*p* = 0.001, OR = 0.83). Plasma transfusions were statistically significantly more frequent in patients without CKD in 2019 (*p* = 0.056, OR 0.88), 2020 (*p* = 0.03, OR = 0.85), 2021 (*p* = 0.01, OR = 0.8), but not in 2022 (*p* = 0.001, OR 1.6). Even though the threshold of statistical significance was reached over this period, PLT transfusions were statistically significantly associated with absence of CKD as follows: 2019 (*p* = 0.01, OR 0.81), 2020 (*p* = 0.001, OR = 0.65), 2021 (*p* = 0.001, OR 0.64), and 2022 (*p* = 0.001, OR 0.49).

PRBC transfusions were associated with the presence of CKD in the first three years, but not in 2022. Conversely, plasma transfusions were administered more frequently to patients without CKD in 2019, 2021, and 2022. 

**Figure 3:**
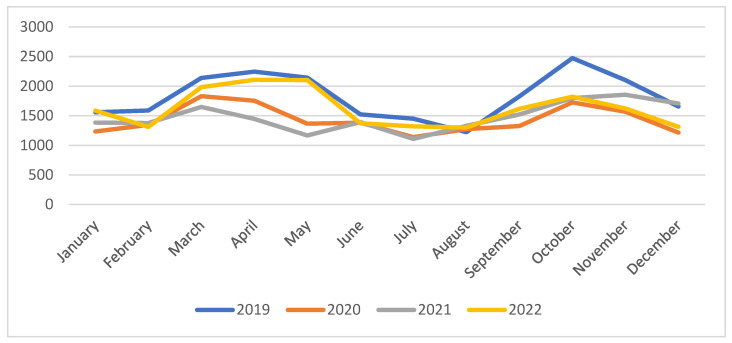
The number of transfused blood units per month, during 2019–2022.

We monitored the number of transfused blood units per month during 2019–2022 and the results are presented in Figure 3.

In January 2019, a number of 1559 units were transfused, then with a decrease in the pandemic year in 2020, with 325 fewer transfusion units compared to 2019. In 2021 we observed a slight increase in transfusion units, and in 2022, the transfused units increased by 202 units compared to 2021.

An increased number of units was noticed in March 2019, namely 2137 units; in 2020, the number of units decreased by 305 units; in 2021, the same with a decrease of 183 units; and for 2022, the number of units had a difference of 334 transfusion units.

Likewise, April 2019 had an increase in transfusion units, namely 2245 units. On the other hand, in 2020, there was a decrease to 1175, and we also observed a decrease in 2021. In 2022, the number of units was almost equal to 2019, with a number of 2109 transfusion units.

In May 2019, the number of units was almost constant at 2143. In 2020, we also observed a decrease to 389 units, and in 2021, there were 1167 transfusion units, and for 2022, we observed an increase in transfused units to 2103 units.

For all years, we notice a slight decrease in transfusion units during the summer months (June, July, August) compared to September, which had 1827 units. In September 2021, there was an increase of 192 transfusion units compared to August, and in 2022, 1617 units were transfused in September, with 1301 units transfused in August.

In October, we could observe an increasing tendency, followed by a sudden decrease in transfusion units between November and December 2019, with 447 units. In 2020, we also observed a decrease in the number of units between November and December. However, in 2021, a number of 1856 units was transfused in November, and a number of 1707 units in December. For the year 2022, in November and December, we also saw a decrease between these months: in November, there were a number of 1622 units, and in December, it decreased by 310 transfusion units.

**Figure 4:**
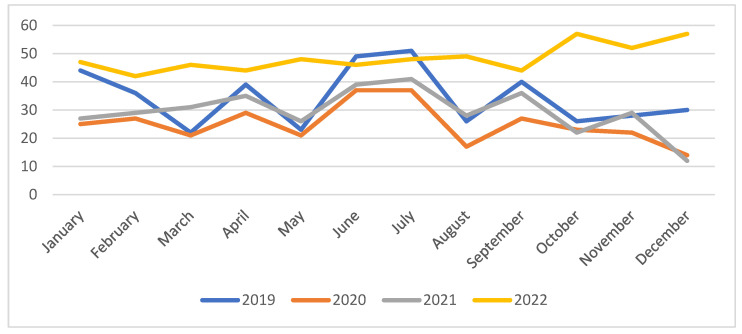
Total number of CKD patients transfused per month.

Regarding the total number of CKD patients transfused monthly in our hospital we could observe the tendency described in Figure 4.

In January 2019, forty-four blood transfusions were performed on patients with chronic kidney disease, totaling 11%, compared to the same period in 2020 (25 patients—8%) and 2021 (27 patients—8%). The number of patients increased significantly in January 2022, reaching 47 patients, maintaining a percentage of 8%.

No significant differences were noted for February in all studied years, but March 2019 showed an impressive decrease in patients with CKD who required blood transfusions, thus reaching 22 patients (5%), compared to February 2019 when 36 cases were registered. A decrease was also noted in March 2020, reaching a number of 21 patients (7%). In 2021, March registered 31 patients (9%). The increase in the number of patients with CKD who required blood transfusion remained high in March 2022 as well, with 46 patients (8%), positioning themselves detachedly ahead of the months of March of previous years.

In April, we are juggling near the same figures, with slight changes compared to March, so in 2019, we registered 39 patients—9%; in 2020, 29 patients and 10%; and in 2021, we reached 35 patients, the highest number recorded so far in 2021, representing 10% of the total patients registered with CKD. The year 2022, as observed previsouly, still remained at a number of 44 patients—8%, and in the future, the number of blood transfusions will be constantly increasing, managing to end the year at its peak.

For the month of May, there was a remarkable decrease in both cases and the percentage of patients for the years 2019, 2020, and 2021 compared to the month previously documented/interpreted, so that we have registered 23 transfusions—6% for 2019, 21 cases and 7% for 2020, as well as 26 patients in need of blood transfusion, i.e., 7% in 2021. The year 2022, as mentioned before, in an upward trend managed to reach 48 patients, with maintenance since March at the percentage of 8%.

The month of June from the period 2019–2022 surprises us with a striking decompensation of patients with CKD, with significantly more of them requiring blood transfusions compared to the months of May in the same years, 2022 continuing to remain at the same absolute and percentage values.

In 2019, July was at the top of the statistics, managing to register the highest number of patients (51) and percentage (12%) that year, figures that were only exceeded in 2022. July 2020 had the same figures as June, with 37 patients and 12%. There was a slight increase in July 2021 compared to June with 41 patients—12%. The year 2022 maintained at 8%, representing a total of 48 patients.

Thus, according to the statistics, the month of July from 2019–2021 had the most numerous cases of transfused CKD patients, and then, in the second half of these years, the cases decreased, with the lowest absolute and percentage values of our statistics recorded for December 2021 and 2022.

Thus, August 2019 showed a decrease, with 26 patients—6%; August 2020, 17 patients with transfusion need representing 6%; 2021 had 28 cases and 8%, and an increase in August 2022, with 49 patients and 8%. In September, 40 patients (10%) required blood transfusion in 2019, 27 patients (9%) in 2020, and a total of 36 patients (10%) in 2021. September 2022 continued to record some of the highest values, namely 44 patients (8%).

We can now mention the maintenance of the percentage of 8% throughout the period March–September 2022. Maintaining the percentage of 8% in 2022 shows us a significant increase in CKD cases compared to 2020 and 2021, with the need for blood transfusions directly proportional to the increase in cases.

The end of 2019 remained at an average number of cases registered throughout the year, not being at any of the extremes, the percentage instead decreasing from 10% in September to 6% in October and remaining at 7% in November and December. The cases themselves show a minimal decrease compared to September, when we registered 40 transfusions; we had 26 patients in October, 28 patients in November, and 30 patients with CKD in December 2019 with transfusion needs. Towards the end of 2020 and 2021, the total number of cases decreased compared to September and to our general statistics, so that, in October 2020, we registered 23 patients (8%), and in 2021, 22 patients (6%).

At the end of 2022, however, the number of cases constantly increased and recorded the most numerous requests for blood transfusions in the period 2019–2022, the percentage undergoing minor changes. October 2022 reached the threshold of 57 patients in need of blood transfusion (10%), a difference of 2% and 13 patients compared to 2022. December 2022 rose back to 57 cases of patients with CKD requiring blood transfusion and 10%.

The pandemic years 2020 and 2021 are characterized by a low number of CKD patients requiring blood transfusion, with lowest transfusion rates in October, corresponding to the highest rates of COVID-19 cases and then, post-pandemic, more cases of CKD appeared (according to the percentage maintenance of 2022 in correlation with the increase in the number of cases) in association with the intra-infectious worsening of the primary renal diseases.

**Figure 5:**
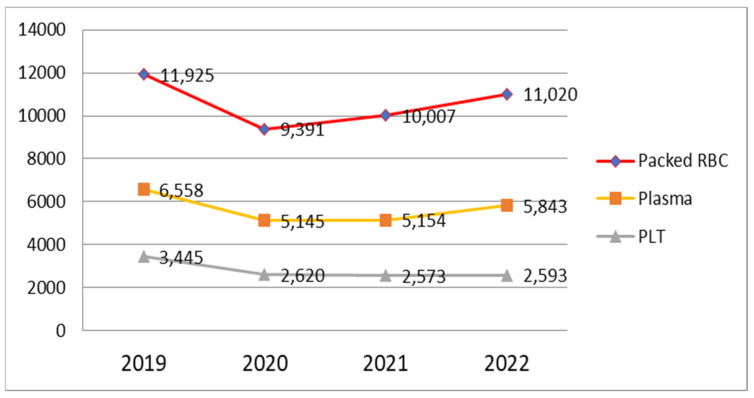
The total number of blood units of each type administered during 2019–2022.

As observed in Figure 5, a number of 11,925 units of erythrocyte concentrate were administered in 2019, then 9391 units in 2020, 10,007 units in 2021, and 11,020 units administered in 2022. In 2019, the number of units of erythrocyte concentrate was 6558, with a decrease in the number of units of fresh frozen plasma to 5145 in 2020. We observed a similar number of PRBC units in 2021 and a slightly increased number of 5843 units administered in 2022.

The number of platelet units in 2019 was 3445, then 2620 units in 2020, and reduced to 2573 units in 2021, with an almost equal value in 2022.

From the total number of blood products administered, there was a constant increase, especially in the need for packed red blood cells (PRBC) and platelet concentrate, with the same trend during the COVID-19 pandemic period, followed by a slight increase in 2021 and 2022 (Figure 5).

The consumption of fresh frozen plasma steadily decreased until 2021, followed by a slight increase in 2022. However, it did not reach the quantity administered before the COVID-19 pandemic.

**Figure 6:**
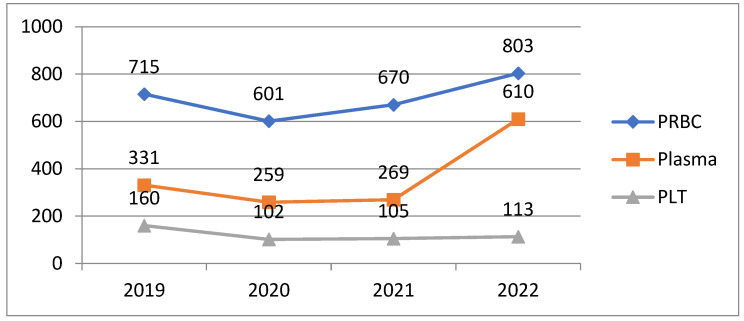
Evolution on transfused blood products in CKD patients, 2019–2022.

After calculating the quantities and type of transfused blood products in CKD patients, between 2019 and 2022, we could establish the evolutive tendency, as presented in Figure 6.

In 2019, 715 units of erythrocyte concentrate were administered. A decrease was observed in the pandemic year 2020, with 601 units administered, increasing to 670 units in 2021, and further increasing to 803 units of erythrocyte concentrate in 2022.

The number of fresh frozen plasma units was 331 in 2019, with a decrease in 2020 to a total of 259 units, with a similar demand in 2021, and an increase to 610 units in 2022.

The number of platelet units administered was 160 in 2019, decreasing to 102 in 2020, remaining almost the same in 2021—106 units, and finally, 113 platelet units were administered in 2022.

## 4. Discussion

One of the significant aspects of healthcare systems influenced by the COVID-19 pandemic has been ensuring adequate supplies of blood and blood-derivatives, including packed red blood cells (PRBC), plasma, and platelets, essential products for managing severe hematological abnormalities in patients with kidney failure [13,21]. The COVID-19 pandemic has impacted blood donation in a variety of ways, and the decrease in the number of blood donors due to mobility restrictions, fear of contracting COVID-19 in public places, or even postponing due to flu-like symptoms has led to a decrease in the production of blood components [21]. The blockade of activities and fear of virus transmission have affected public health and the economy, causing a significant decrease in the number of blood donations worldwide [22,23].

In our research, we noticed that the highest level of transfused blood components was recorded in the pre-pandemic year 2019, with 22,722 units and the highest number of transfused patients, 6158, for all the hospital wards. This volume of transfusions was not overpassed in 2022, after the pandemics.

During the pandemic years, as expected, in the strict, restrictive conditions imposed by the repeated lockdowns [24], the number of transfused patients and transfused blood units decreased dramatically, by 4% and 7%, respectively, with no significant differences between 2020 and 2021. In CKD patients, the number of transfused patients and transfused blood units decreased by 5% and 7%, respectively. The global demand for blood products, mainly PRBC, FFP, and PLT, decreased in 2020 due to reduced hospitalizations for non-COVID patients and the suspension of elective surgeries. However, in 2021 and 2022, COVID wards were established in multiple hospital departments for patients with multiple comorbidities.

It is interesting to notice that, unlike the general trend of transfusion activity in the entire hospital, there were more patients with CKD transfused with PRBC and plasma in 2022 than before the pandemic, and the number of blood units was higher in 2022 than in 2019 for these two blood products.

The pandemic years 2020 and 2021 are characterized by a lower number of CKD patients requiring blood transfusion, with lowest transfusion rates in October, corresponding to the highest rates of COVID-19 cases.

According to a study published in 2022, the number of blood donors decreased in the following years, highlighting a general decrease in blood availability, ranging from 10% to 50% [25].

In the pandemic years, a significant decrease in the number of patients and transfused blood products was observed, in line with studies indicating a significant reduction in donations compared to 2019 [26]. The main challenge encountered was engaging COVID-19-recovered patients in the blood donation process and lack of knowledge regarding the potential risk of SARS-CoV-2 transmission via blood derivatives. Additionally, blood collection centers had to undergo thorough disinfection, and medical waste disposal needed to be managed more meticulously [27].

The coping strategies adopted by different countries to maintain a safe and uninterrupted blood transfusion chain at the blood bank have provided valuable lessons for future preparedness in facing similar situations [28,29,30,31,32,33,34].

CKD patients were found to be more susceptible to severe forms of COVID-19 in all studies performed during pandemics and after it was officially ended. In a Portuguese study that collected data from two databases: (1) the nationwide database of all confirmed COVID-19 cases in Portugal (n = 20,293); and (2) the community-based COVID-19 Barometer survey, containing data on health status, perceptions, and behaviors during the first wave of COVID-19 (n = 171,087), the researchers assessed the association between relevant chronic diseases (i.e., respiratory, cardiovascular, and renal diseases; diabetes; and cancer) and death, especially associated with intensive care unit (ICU) admission, associated with COVID-19 infection [35].

A special group of CKD patients affected by COVID-19 pandemics were those with kidney transplantation. Current data indicates that kidney transplanted patients, due to their immunosuppressive status, are more vulnerable to severe infections. Also, the clinical evolution and outcome of the COVID-19 disease in renal graft recipients are heterogeneous and usually more severe than in the general population, with high risk of severe complications and death [36,37]. Our study group included 24 kidney transplant recipients that received mainly erythrocyte concentrate, 66.6% were COVID-19 positive during their hospitalization, two of them lost their graft and switched to hemodialysis and one patient died because of cardio-respiratory insufficiency due to severe viral infection.

The ESKD dialysis patients were directly and indirectly affected by COVID-19 pandemics. The necessity to implement social distancing for patients requiring dialysis during the pandemic prompted medical authorities to publish strict recommendations regarding safety measures to reduce the risk of SARS-CoV-2 transmission and optimize dialysis treatment during the COVID-19 pandemic, but with a lot of medical, psychological and social effects, reducing the quality of life of these patients [38]. Most hemodialysis patients have an increased risk of thrombosis, which has led to the recommendations to reduce the target hemoglobin level. However, this must be balanced with the need to avoid unnecessary blood transfusions with considerations regarding the severity of COVID-19 and the risk of human leukocyte antigen (HLA) sensitization after blood transfusion and, thus, drastically reducing chance for these patients to receive a kidney graft in the future [39,40].

Regarding the total number of transfused patients from our study group, which was 6158 in 2019, there was a sharp decrease in 2020, with the onset of the COVID-19 pandemic and hospitalization restrictions (5255 patients]. The COVID-19 pandemic exacerbated the blood supply shortage; blood consumption decreased especially for non-emergency surgical interventions, including postoperative transfusions worldwide [41]. Studies have indicated that hospitalized COVID-19 patients require much fewer blood transfusions compared to other hospitalized patients, suggesting that COVID-19 is not usually associated with severe hemorrhagic conditions requiring frequent blood transfusions [42,43]. In 2021, the number of transfusions slightly increased to 5979, and in 2022, the total number of transfused patients (regardless of the type of blood component] was 6704 patients. The average number of blood products administered per patient in our analysis showed a slight decrease throughout the pandemic period. On the other hand, a decrease in the number of transfused patients was observed throughout 2020 compared to the average of the previous years, including the first three months when COVID-19 was not a concern. This decrease was also maintained in 2021–2022, as confirmed by other studies [44,45,46].

From the total of labile blood products administered, a constant increase was observed, especially concerning the need for packed red blood cells (PRBC]. The complex situations generated by the pandemic have led to a significant decrease in red blood cell (PRBC) transfusions [47].

Compared to 2019, 2020 began with a lower number of requests for blood units, and after the initiation of the pandemic lockdown, this number continued to decrease in August 2020. In total, in 2020 and 2021, fewer requests for blood units were recorded compared to 2019. The number of requests for blood units in 2020 was significantly lower than that in 2019, affecting critical wards, like general surgery and even cardiothoracic surgery services [48,49].

As the pandemic progressed, institutional adherence to transfusion decisions improved for RBC transfusion units compared to trends from the previous year [50]. However, in our study, a similar improvement was not observed regarding platelet or plasma transfusions. The total number of blood units issued in 2021 was significantly lower compared to 2019 and was higher but statistically nonsignificant compared to 2020. Similarly, the total number of blood units issued in 2022 was significantly higher than in 2020 and 2021 and lower but statistically nonsignificant compared to 2019.

The present study recorded a consistent increase, especially in the demand for packed red blood cells and platelet concentrates, with the same trend during the COVID-19 pandemic period: a sharp decrease in 2020 followed by a slight increase in 2021 and 2022 (see Figure 4).

Studies conducted during COVID-19 pandemic indicate a significant decrease in both whole blood and platelet concentrate donations, with a range of 5% to 86% for whole blood and 3% to 34% for platelet concentrates. This trend was particularly noticeable in February 2020. Additionally, the supply and utilization of both packed red blood cells (PRBC] and fresh frozen plasma (FFP] have decreased, with a range of 4% to 40% for PRBC and 9% to 58% for FFP. Compared to 2019, there has been a decrease in blood transfusions in surgery, while their usage in emergency departments and internal medicine treatments has increased [51].

The COVID-19 pandemic has accelerated renal pathology and worsened outcomes for patients with kidney conditions. Patients’ access to short-term hospitalization has been reduced to minimize exposure and infection risk. Consequently, the need for emergency dialysis has significantly increased, including continuous renal replacement therapy (CRRT] for critically ill patients from ICU, who experienced acute-on-chronic kidney disease, with a brutal crush of the chronic, but stable, previously altered kidney function [51,52]. More patients associated the initiation of CRRT or chronic dialysis with a process that may be associated with significant pain and discomfort, placing pain management at the center of many studies [53].

Chronic kidney disease confers a higher risk of mortality for patients admitted to medical wards, and for those admitted to intensive care units, with a special evolution of the disease, acting like a severe acute episode on a previously chronic, apparently stable kidney disease. Mortality increases with the number of RBC units transfused [49,54]. Between 2020–2022, from the total number of transfused patients in our study, 254 with CKD patients (16%) and 798 non-CKD (4%) died in-hospital.

A retrospective study conducted in two emergency hospitals from Romania aimed to describe the influence of COVID-19-related factors on the severity, outcome and timing of acute kidney injury (AKI), one of the most severe complications of SARS-CoV-2 infection. A total of 268 adult patients were included in the study (average age 72.28 years; 169 were men); 157 had AKI and 111 had acute-on-chronic-kidney disease (ACKD). Recovery of renal function was considered total when serum creatinine levels returned to normal values (in AKI patients) or when it returned to baseline values before admission (in ACKD patients). From the 111 patients with ACKD, 19 (17%) required CRRT and needed blood transfusions [55].

Strengths and Limitations: our study has several limitations. First, it was a single center, retrospective study. In addition, the sample size was relatively small. Laboratory data were not uniformly available for every patient, so we could not perform dynamic measurement of hemoglobin level. The clinical indications for transfusion were not available for all patients so we could not investigate the clinical reasons for perioperative anemia in surgical CKD patients.

## 5. Conclusions

The impact of the COVID-19 pandemic on the blood transfusion program within our emergency county medical unit in South-eastern Romania has been particularly significant, as evidenced by the evaluation of the total number of blood products administered between 2019 and 2022, as well as the assessment of the number of transfused patients during this time.

During the pandemic years, as expected, the number of transfused patients and transfused blood units decreased by 4% and 7% respectively, with no significant differences between 2020 and 2021. In CKD patients, the number of transfused patients and transfused blood units decreased by 5% and 7%, respectively, with a mean of 3 units/patient (range 1–10).

In 2019, the year before the pandemic, there was the highest number of administered blood products: 22,722 units and the highest number of transfused patients: 6158 for all the hospital wards. These transfusion volumes were not overpassed in 2022, after the pandemic. Unlike the general trend of transfusion activity, there were more patients with CKD transfused in 2022 (580) than before pandemic (414 in 2019), and the number of blood units was higher in 2022 than in 2019 for PRBC and plasma.

The pandemic years 2020 and 2021 were characterized by a reduced number of patients with CKD who required blood transfusion, because of dramatically limitations of hospitalizations for chronic patients, with lowest transfusion rates in October, corresponding to the highest rates of COVID-19 cases.

The adaptive strategies implemented to ensure the continuity and safety of the blood transfusions during the COVID-19 pandemic included mainly restrictive transfusion and limitation of elective surgical procedures. This has provided valuable insights for the management of future similar situations, thereby strengthening the resilience of the blood transfusion system in the face of challenges that healthcare workers will encounter in future pandemics. For CKD patients, implementing the latest transfusion decisions and providing the necessary blood transfusions were essential for the proper management of these patients, with high risk of alloimmunization and lost chance for a future kidney transplant.

## Figures and Tables

**Table 1:** Demographic and clinical characteristics of the study group (n = 1590).

Demographic Features	
Age (mean ± standard deviation)	62.8 ± 18.7
Gender, *n* (%)	
Female	62.2
Male	37.8
Urban area *n* (%)	52.3
Clinical features	No. of patients (%)
*CKD stage* (eGFR CKD-EPI)	
G3 (30–59 mL/min/1.73 m^2^)	711 (44.7)
G4 (15–29 mL/min/1.73 m^2^)	455 (28.6)
G5 (<15 mL/min/1.73 m^2^)	249 (15.7)
G5-HD	296 (18.6)
G5-PD	15 (0.7)
G 3-5 T	24 (1.5)
*Diabetes mellitus*	762 (47.9)
*Smokers*	368 (23.1)
*Arterial hypertension*	1012 (63.6)
*Hematological abnormalities* (*n*)	
Anemia	1068
Thrombocytopenia	72
Clotting disturbances	509
*Use of drugs in diagnosis*	
Antiplatelets	821 (51.6)
Anticoagulants	216 (13.5)
NSAIDs	402 (25.2)
*SARS-CoV-2 positive*	588 (36.9)
*Admission Clinic/Department*	
Nephrology	889 (55.9)
Urology	191 (12)
General Surgery	121 (7.6)
Cardiovascular surgery	119 (7.4)
Gynecology	107 (6.7)
Cardiology	91 (6.2)
Rheumatology	72 (4.5)

CKD—chronic kidney disease; HD—hemodialysis; PD—peritoneal dialysis; NSAIDs: non-steroidal anti-inflammatory drugs.

**Table 2:** The number of blood transfusions and components of blood per year in study groups.

Year	Total No. of Transfused Blood Units (Mean ± SD)	No. of PRBC Units		No. of FFP Units		No. of PLT Units		No. of Transfused Blood Units in CKD Patients
		Total	With CKD	Total	With CKD	Total	With CKD	
2019	21,928 (1827 ± 384)	11,925	715	6558	331	3445	160	1206
2020	17,156 (1430 ± 232)	9391	601	5145	259	2620	102	962
2021	17,734 (1478 ± 236)	10,007	670	5154	269	2573	105	1044
2022	19,456 (1621 ± 315)	11,020	803	5843	610	2593	113	1526

**Table 3:** The total number of CKD patients transfused with different blood products in 2019–2022 period.

Year	Total No. of Patients (Mean ± SD)	Patients Transfused with PRBC	Patients Transfused with Plasma	Patients Transfused with PLT
2019	414 (35 ± 10)	283	107	24
2020	300 (25 ± 7)	211	74	15
2021	355 (30 ± 7,9)	254	84	17
2022	580 (48 ± 4,8)	320	244	16

**Table 4:** Group differences regarding PRBC transfusion units in patients with CKD and without CKD.

Year	Parameter	Total Blood Units	No of Transfusion Units in Patients with CKD	No of Transfusion Units in Patients without CKD	*p*-Value	OR
2019	PRBC	11,925	715	11,210	**0.004**	1.23
	Plasma	6558	331	6227	**0.056**	0.88
	PLT	3445	160	3285	**0.01**	0.81
2020	PRBC	9391	601	8790	**0.001**	1.4
	Plasma	5145	259	4886	**0.03**	0.85
	PLT	2620	102	2518	**0.001**	0.65
2021	PRBC	10,007	670	9337	**0.001**	1.41
	Plasma	5154	269	4876	**0.01**	0.8
	PLT	2573	105	2468	**0.001**	0.64
2022	PRBC	11,020	803	11,020	**0.001**	0.83
	Plasma	5843	610	5233	**0.001**	1.6
	PLT	2593	113	2480	**0.001**	0.49

## Data Availability

Data unavailable due to privacy restrictions.
